# Metabolic cost of osmoregulation by the gastro-intestinal tract in marine teleost fish

**DOI:** 10.3389/fphys.2023.1163153

**Published:** 2023-04-26

**Authors:** A. Little, C. Pasparakis, J. Stieglitz, M. Grosell

**Affiliations:** ^1^ University of Miami’s Rosenstiel School of Marine, Atmospheric and Earth Science, Miami, FL, United States; ^2^ Department of Biology, McMaster University, Hamilton, ON, Canada; ^3^ Bodega Marine Laboratory, University of California Davis, Bodega Bay, CA, United States

**Keywords:** intestinal ion absorption, esophagus, water absorption, tissue respirometry, standard metabolic rate

## Abstract

**Introduction:** Although dozens of studies have attempted to determine the metabolic cost of osmoregulation, mainly by comparing standard metabolic rates (SMR) in fish acclimated to different salinities, consensus is still lacking.

**Methods:** In the present study, using the Gulf toadfish, *Opsanus beta*, we aimed to determine the metabolic cost of esophageal and intestinal osmoregulatory processes by estimating ATP consumption from known ion transport rates and pathways and comparing these estimates with measurements on isolated tissues. Further, we performed whole animal respirometry on fish acclimated to 9, 34 and 60 ppt.

**Results and Discussion:** Our theoretical estimates of esophageal and intestinal osmoregulatory costs were in close agreement with direct measurements on isolated tissues and suggest that osmoregulation by these tissues amounts to ∼2.5% of SMR. This value agrees well with an earlier attempt to estimate osmoregulation cost from ion transport rates and combined with published measurements of gill osmoregulatory costs suggests that whole animal costs of osmoregulation in marine teleosts is ∼7.5% of SMR. As in many previous studies, our whole animal measurements were variable between fish and did not seem suited to determine osmoregulatory costs. While the esophagus showed constant metabolic rate regardless of acclimation salinity, the intestine of fish acclimated to higher salinities showed elevated metabolic rates. The esophagus and the intestine had 2.1 and 3.2-fold higher metabolic rates than corresponding whole animal mass specific rates, respectively. The intestinal tissue displays at least four different Cl^−^ uptake pathways of which the Na^+^:Cl^−^:2 K^+^ (NKCC) pathway accounts for 95% of the Cl^−^ uptake and is the most energy efficient. The remaining pathways are *via* apical anion exchange and seem to primarily serve luminal alkalinization and the formation of intestinal CaCO_3_ which is essential for water absorption.

## 1 Introduction

Marine teleost fish live in a strongly desiccating environment as they maintain their internal osmotic pressure (310–350 mOsm) much below that of their surroundings. The osmoregulatory process facilitating marine teleost survival has been the subject of study for nearly a century (Smith, 1930) and more recent efforts have aimed to determine the metabolic cost of this vital process. While we have a good understanding of many physiological processes involved in salt and water balance, the question of metabolic cost remains to be answered with any degree of certainty.

Marine teleost fish drink seawater to compensate for diffusive water loss (Smith, 1930). Early studies found that ingested seawater is desalinized by the water-impermeable esophagus and that solute-coupled water absorption occurs in the intestine with excretion of the excess Na^+^ and Cl^−^ gain across the gill (Shehadeh and Gordon, 1969; Parmelee and Renfro, 1983). The stomach of unfed fish likely plays little, if any, role in osmoregulation (Larsen et al., 2014). More recent studies have revealed that alkalinization of the intestinal lumen is essential for precipitation of CaCO_3_ (ichthyocarbonates) and thereby reduction in luminal osmotic pressure to promote water absorption (Wilson et al., 2002; Grosell et al., 2009b; Genz et al., 2011). Although the intestine is largely impermeable to divalent ions, some are assimilated and ultimately cleared by the kidney in low volumes of isosmotic urine (reviewed in (Larsen et al., 2014)).

The esophageal and intestinal osmoregulatory processes are both ATP–demanding, with basolateral Na^+^/K^+^ pumps and apical proton pumps (in the intestine) facilitating Na^+^ and Cl^−^ uptake (Guffey et al., 2011). Several studies have demonstrated upregulation of intestinal Na^+^/K^+^ pumps as well as apical proton pumps as fish acclimate to higher salinities (Jampol and Epstein, 1970; Colin et al., 1985; Madsen et al., 1994; Fuentes et al., 1997; Kelly et al., 1999; Seidelin et al., 2000; Guffey et al., 2011), suggesting increased metabolic demands in these tissues. Recent and elegant studies have demonstrated increased blood flow to the gastro-intestinal tract of seawater compared to freshwater-acclimated rainbow trout, supporting the suggestion that osmoregulation in hyperosmotic environments imposes a metabolic demand on the intestinal tissue (Brijs et al., 2015; Brijs et al., 2016). However, no study to date has directly determined the metabolic costs of esophageal and intestinal osmoregulatory processes.

Metabolic demands of osmoregulation have been the subject of much interest for decades, with dozens of papers published on the subject (reviewed in (Ern et al., 2014)). Most of these studies have attempted to determine the cost of osmoregulation from differences in whole animal metabolic rates (oxygen consumption) between fish acclimated to freshwater, intermediate salinities, and seawater. Generally, the expectation was that fish held in salinities near that of their body fluids would display limited osmoregulatory costs and lower standard metabolic rates (SMRs; metabolic rate in fully resting, unfed fish acclimated to their environment). The differences between oxygen consumption invoked by freshwater or seawater acclimation, compared to those at isosmotic salinities, are thought to reflect the metabolic costs of osmoregulation at those salinities. In short, there is no consensus in findings among these studies and estimates of osmoregulatory costs vary from few % to >30% of standard metabolic rate (Ern et al., 2014; Christensen et al., 2018; Christensen et al., 2019). While interspecies differences may account for some of this variation, interindividual differences in non-osmoregulatory organismal responses, such as spontaneous activity and/or stress, likely also contribute to the lack of consensus in the field (Chabot et al., 2016).

Surprisingly, a second approach of using isolated osmoregulatory tissues to estimate the metabolic costs of osmoregulation has not been frequently employed, even though it avoids the problems of confounding organismal responses. One study using isolated branchial arches from cutthroat trout (*Oncorhynchus clarki*) and blockers of relevant ATPases determined the branchial cost of osmoregulation to be 3.9% and 2.4% of BMR following acclimation to freshwater and seawater, respectively (Morgan and Iwama, 1999). A third approach to estimate osmoregulatory metabolic costs is utilizing known ion transport rates and ATP demands per mole of ions transported, which offers the same advantages as measurements of metabolic rates on isolated tissues. This approach has also only been applied once and the estimated metabolic cost of osmoregulation was in the order of 7%–17% of SMR in marine teleosts (Kirschner, 1993).

To date, no attempts have been made to measure metabolic rates in the esophagus of teleost fish, to estimate metabolic costs based on ion transport rates for this tissue, or to measure the metabolic cost of intestinal transport processes related to osmoregulation. In the present study, we aimed to investigate the metabolic costs of osmoregulation by comparing the three approaches discussed above; whole animal respirometry, isolated tissue respirometry, and metabolic estimates calculated from known ion transport rates. We performed whole animal and isolated-tissue (esophagus and intestine) respirometry in gulf toadfish (*Opsanus beta*) acclimated to 9, 34 and 60 ppt. In addition, we took advantage of the available literature on ion transport across the esophageal and intestinal tissue of this teleost to compare predicted osmoregulatory costs across the three methods. The two latter methods showed strong agreement and are supported by earlier estimates of osmoregulatory costs in the intestinal tracts of other marine teleosts (Kirschner, 1993).

## 2 Methods

### 2.1 Animal husbandry

Gulf toadfish (*Obsanus beta*) were obtained from commercial shrimp fishermen trawling Biscayne Bay, Miami, from October 2016 to January 2017. On arrival, toadfish were treated for ecto-parasites (McDonald and Crosell. 2006) and sorted by size into 40-L tanks (8–10 per tank) with aerated, sand-filtered, flow-through seawater from Bear Cut (21°C–26°C, 30–35 ppt salinity). Pieces of polyvinylchloride tubing were used as shelters to reduce stress and decrease aggressive behavior. Toadfish were fed squid to satiation weekly but fasted for at least 144 h before experimentation. Toadfish were held in ambient seawater for at least 2 weeks prior to experimental treatments. All general animal care and animal sacrifice protocols were carried out in accordance with relevant guidelines for experiments on teleost provided by University of Miami IACUC (Institutional Animal Care and Use Committee) and experimental protocols were approved by University of Miami IACUC (15–019). University of Miami’s IACUC is accredited by the Association for Assessment and Accreditation of Laboratory Animal Care (AALAC). Toadfish were collected with the approval and in accordance with guidelines outlined by the Florida Fish and Wildlife Conservation Commission (SAL-16-0729-SR).

#### 2.1.1 Salinity acclimation

Toadfish (range: 24.0–43.7 g for whole-animal respirometry; 25–45 g for intestinal preps; and 54–130 g for esophageal preps) were housed in 20-L tanks with aerated, sand-filtered seawater adjusted to 9 ppt (seawater diluted with reverse osmosis purified water), 34 ppt (seawater), or 60 ppt (seawater supplemented with Instant Ocean marine salt, Spectrum Brands, Blacksburg, VA, United States of America) for a minimum of 10 days. Common to all experiments, toadfish assigned to the 9 ppt acclimation groups were introduced to 20 ppt seawater day 1, followed by 17.5 ppt day 3, 15 ppt day 5, 12.5 ppt day 7, and 9 ppt day 9. Toadfish assigned to 34 ppt and 60 ppt acclimation treatments were transferred immediately to their respective salinities. Tank water was maintained at 23˚C ± 1°C with 75% tank water changed every second day. Toadfish were maintained at their target salinity for a minimum of 10 days and were fasted for 6–13 days prior to experimental procedures to avoid influence of feeding status on metabolic rates.

#### 2.1.2 Whole-animal respirometry

Toadfish (n = 10–14) were housed individually in 20 L tanks with aerated, sand-filtered seawater at 22°C. Following acclimation to constant salinity, intermittent flow respirometry was performed using Brett-style swim respirometers (Loligo Systems, Denmark) as previously described (Mager et al., 2014; Stieglitz et al., 2016) but with constant and low water velocity to ensure adequate mixing (< 0.5 body lengths per second). Fish were sedentary on the bottom under these conditions. SMR was determined by fitting a double Gaussian curve to *Ṁ*O_2_ measurements with an *r* ^2^ > 0.80. Elevated values of *Ṁ*O_2_ were excluded using the first (higher) normal distribution, whereas the second normal distribution was used as the best estimate of SMR (Steffensen et al., 1994; Herskin, 1999; Jordan and Steffensen, 2007; Svendsen et al., 2012).

#### 2.1.3 Theoretical calculations for MO_2_ in esophageal and intestinal epithelia

Gulf toadfish held in seawater display drinking rates of 2.6 mL kg^-1^ h^-1^ (Genz et al., 2008) which corresponds to an intake of 1,271 µmol Cl^−^ kg^-1^ h^-1^ (Grosell, 2014). Assuming no water absorption by the esophagus (Hirano and Mayer-Gostan, 1976; Parmelee and Renfro, 1983) and an average gastric Cl^−^ concentration in unfed marine teleosts fish of 268 mM (n = 11) (Grosell, 2014), esophageal Cl^−^ absorption can be estimated to be (1,271 µmol Cl^−^ kg^-1^ h^-1^—(2.6 mL^-1^ kg^-1^ h-1 X 268 mM Cl^−^)) 575 µmol Cl^−^ kg^-1^ h^-1^ leaving 697 µmol Cl^−^ kg^-1^ h^-1^ to be transferred through the pyloric sphincter into the anterior intestine (Table 1). Rectal fluids voided at low rates contain little Cl^−^, resulting in a limited rectal excretion of 63 µmol Cl^−^ kg^-1^ h^-1^ (Genz et al., 2008), which is a product of an intestinal absorption of (697–63 µmol Cl^−^ kg^-1^ h^-1^) 634 µmol Cl^−^ kg^-1^ h^-1^ (Table 1).

**TABLE 1:** Esophageal and intestinal ion transport pathways and their contribution to Cl^−^ uptake, ATP and O_2_ consumption as well as their relative contribution to standard metabolic rate (SMR). See text for further detail.

Route of Cl^−^ uptake	Cl^−^ absorption (µmol kg^-1^ h^-1^)	Fraction of total (%)	ATP:Cl^−^	ATP consumed (µmol kg^-1^ h^-1^)	O_2_ consumed (µmol kg^-1^ h^-1^)	Fraction of SMR (%)
Esophageal absorption						
NHE2-AE mediated	575	100	0.33	94.8	19.0	1.22
Intestinal absorption						
NBC mediated	17.0	2.7	0.22	3.7	0.75	0.04
CAc-NHE mediated	15.3	2.4	0.66	10.1	2.02	0.12
CAc-VHA mediated	1.7	0.3	0.5	1.7	0.34	0.02
NKCC	599.8	94.6	0.17	102	20.39	1.21
Total	633.8	100		117.5	23.5	1.40

Cl^−^ (and Na^+^) is absorbed across the esophageal epithelium by both passive (paracellular) and active (transcellular) paths of approximate equal quantities in the flounder (*Pseudopleuronectes americanus*) (Parmelee and Renfro, 1983). Assuming the same is true for toadfish, active absorption of (575/2) 287 µmol Cl^−^ kg^-1^ h^-1^ takes place across the esophageal epithelium. For toadfish, the active esophageal salt absorption is mediated by apical anion and Na^+^/H^+^ (NHE2) exchangers deriving energy for H^+^ extrusion and Na^+^ uptake from the basolateral Na^+^/K^+^-ATPase (Esbaugh and Grosell, 2014). Assuming an active 1:1 C l^-^:Na^+^ absorption rate by the esophageal epithelium and a 3Na^+^/ATP stoichiometry of the Na^+^/K^+^-ATPase (NKA), Cl^−^ absorption can be estimated to occur at the cost of 0.33 mol ATP/mol Cl^−^. A further assumption of 5 mol ATP produced per mol of O_2_ consumed (Hinkle, 2005) allow for estimation of O_2_ consumption arising from esophageal active absorption of NaCl (Table 1).

Multiple Cl^−^ uptake pathways operate in parallel in the intestine of marine teleosts (Grosell, 2006) (Figure 2). In the toadfish, rectal HCO_3_^−^ excretion in the order of 68 μmol kg^-1^ h^-1^ (Genz et al., 2008) allows for quantification of intestinal Cl^−^ uptake by Cl^−^/HCO_3_^−^ exchange. This anion exchange fraction of intestinal Cl^−^ absorption occurs *via* SLC26a6 which is an electrogenic anion exchanger operating by secreting 2 (or more) HCO_3_^−^ ions in exchange for absorption of 1 C l^-^ ion across the apical membrane (Kurita et al., 2008; Grosell et al., 2009b). Assuming a 2:1 HCO_3_^−^/Cl^−^ exchange ratio toadfish intestinal Cl^−^ absorption *via* anion exchange (Kurita et al., 2008; Grosell et al., 2009b) amounts to 5.4% of total intestinal Cl^−^ uptake with the rest (94.6%) attributed to Na^+^/K^+^/2 C l^-^ co-transport *via* apical NKCC2 driven by the basolateral NKA. We have no evidence for the presence of Na^+^:Cl^−^ cotransport in the gulf toadfish intestine but cannot rule out a role for this transporter in other species. Considering the stoichiometry of NKCC2 (2 mol of Cl^−^ absorbed per mole of Na^+^) and NKA (3 mol of Na^+^ transported per mole of ATP consumed), absorption of Cl^−^ via NKCC2 consumes 0.17 mol ATP/mol Cl^−^ absorbed. With this ratio, the intestinal Cl^−^ absorption rate via NKCC2 and the above assumption of ATP production per mol of O_2_ consumed, O_2_ consumption via the NKCC2 Cl^−^ absorption pathway can be estimated (Table 1). We are aware of three distinct pathways associated with Cl^−^ absorption via anion exchange in the toadfish. Approximately 50% of anion exchange is fueled by transepithelial HCO_3_^−^ movement mediated by basolateral Na^+^/HCO_3_^−^ co-transport (NBC) and apical SLC26a6 anion exchange (Grosell and Genz, 2006). The remaining 50% of Cl^−^ absorption mediated by SLC26a6 is fueled by hydration of endogenous CO_2_, a reaction mediated by carbonic anhydrase (CAc) (Grosell and Genz, 2006; Sattin et al., 2010). The CAc mediated Cl^−^ absorption relies on secretion of H^+^ which occurs largely *via* basolateral Na^+^/H^+^ exchange (90% of the CAc mediated Cl^−^ uptake) (Grosell and Genz, 2006) but also *via* apical V-type H^+^ ATPase (VHA) (10% of the CAc mediated Cl^−^ uptake) (Grosell et al., 2009b; Guffey et al., 2011).

Considering first the metabolic cost of the NBC-SLC26a6 pathway, we assumed a stoichiometry of 3 HCO_3_^−^:1Na^+^ (Chang et al., 2012) and the usual 3Na^+^:ATP for NKA and 2HCO_3_^−^:Cl^−^ for SCL26a6. Under these assumptions, the Cl^−^ absorption taking place *via* this pathway occurs at a cost of 0.22 mol of ATP/mol Cl^−^. With the above assumption of 5 mol ATP produced per mol of O_2_ consumed we estimated the oxygen consumption for Cl^−^ absorption attributable to the NBC-SLC26a6 pathway (Table 1). The stoichiometry of the only marine fish NBC transporter examined, the euryhaline pufferfish, *Takifugu obscurus*, is dependent on extracellular Na^+^ and HCO_3_^−^ and appears to be higher than for human NBC (Chang et al., 2012). Since the pufferfish NBC stoichiometry was determined in *xenopus* oocytes, it was not measured under fully physiological conditions but likely falls somewhere between 1.93 and 4.06 at extracellular Na^+^ and HCO_3_^−^ concentrations relevant for marine teleosts (Chang et al., 2012).

Considering next the two carbonic anhydrase (CAc) dependent Cl^−^ absorption pathways, NHE-SCL26a6 and VHA-SLC26a6, we assumed a 1Na^+^:1H^+^ ratio for NHE and a 0.5 mol ATP/mol H^+^ ratio for VHA. Under these assumptions Cl^−^ absorption by NHE-SLC26a6 and VHA-SLCa6 pathways occur at a cost of 0.66 and 1 mol of ATP/mol of Cl^−^, respectively, and again under the assumption of 5 mol ATP produced per mol of O_2,_ oxygen consumption attributable to both CAc-dependent pathways were calculated (Table 1). The stoichiometry of VHA is variable (Tomashek and Brusilow, 2000; Maxson and Grinstein, 2014) but generally assumed to be 2H^+^/ATP, or higher (Tomashek and Brusilow, 2000; Maxson and Grinstein, 2014; Anandakrishnan and Zuckerman, 2017).

### 2.2 Empirical measures of MO_2_ in isolated esophageal and intestinal tissue preparations

Toadfish acclimated to 9 ppt (N = 7 for esophageal; N = 6 for intestinal), 34 ppt (N = 7 for esophageal; N = 6 for intestinal) and 60 ppt (N = 6 for esophageal; N = 6 for intestinal) as described above were sacrificed using a lethal dose of 0.2 g/L MS-222 buffered with 0.4 g/L NaHCO_3_. MO_2_ for isolated anterior intestine and esophagus were measured using a custom-designed epithelial respirometer (Loligo Systems, Tjele, Denmark) described elsewhere (Taylor and Grosell, 2009; Secor et al., 2012). Intestinal and esophageal tissues were dissected, weighed, and mounted so that 0.87 cm^2^ of tissue was exposed to two half-chambers (2.80 mL each), with mucosal saline on the gut side, and serosal saline on the blood-side. Serosal saline compositions (Table 2) were the same between esophageal and intestinal MO_2_ preparations, and were adjusted to pH 7.8 and 330 mmol/kg osmolality with mannitol, and sterile filtered. Mucosal salines for intestinal MO_2_ measurements (Table 2) were made to approximate the composition of anterior intestinal fluids of toadfish acclimated to 9, 34, and 60 ppt (McDonald and Grosell, 2006) and were adjusted to 330 mmol/kg osmolality with mannitol, and sterile filtered. Mucosal solution for esophageal measurements was sterile-filtered seawater of the acclimation salinity. All salines were pre-gassed with air, rather than custom O_2_ mixes, so that MO_2_ could be compared to whole-animal measurements. Note that intestinal O_2_ consumption is not limited by O_2_ levels in air (Taylor and Grosell, 2009).

**TABLE 2:** Saline composition (mM).

	Serosal	Mucosal (9 ppt)	Mucosal (33 ppt)	Mucosal (60 ppt)
NaCl	151	135	69	20
KCl	3	5	5	5
MgSO_4_	0.88	77.5	77.5	80
MgCl2	-	22.5	22.5	50
Na_2_HPO_4_	0.5	-	-	-
KH_2_PO_4_	0.5	-	-	-
CaCl_2_	1	3	3	3
NaHCO_3_	5	-	-	-
HEPES free acid	11	-	-	-
HEPES Na^+^ salt	11	-	-	-
Urea	4.5	-	-	-
-Glucose	5	-	-	-

Salines in half chambers were continuously mixed by micromagnetic glass-coated Teflon stir bars (Loligo Systems), and a Teflon tissue mount ensured that the system was gas-tight (Taylor and Grosell, 2009). Oxygen measurements were conducted using fiber-optic cables secured to the outside walls of glass half-chambers to illuminate a fiber-optic sensor spot glued to the inside wall of each respective half-chamber. Each cable was connected to a separate single-channel oxygen meter (Fibox 3) used in conjunction with Oxy-View software (PST3-V6.02; PreSens, Regensburg, Germany). A standard curve and/or two-point calibrations were used to convert the signal (i.e., phase angle Φ) to oxygen content using salines pre-gassed with air for 100% air saturation and supersaturated with sodium sulfite for 0% oxygen calibration. O_2_ measurements were recorded every second at 22°C ± 1 °C with automatic temperature compensation. Intermittent-flow respirometry was performed to determine oxygen consumption rates of isolated tissue by flushing and replacing salines using a peristaltic pump (WPI Peri-Star). Flush cycles (∼3 min) were optimized for complete saline replacement between closed cycles, and time intervals (∼15 min) during closed measurements were monitored to ensure that O_2_ concentrations did not drop below 80% air saturation. Tissue MO_2_ was calculated from the sum of mucosal and serosal O_2_ consumption rates and normalized to the mass of the exposed tissue. Background respiration rates (blanks) were measured using parafilm to separate the two half-chamber respirometers and were found to be negligible.

### 2.4 Statistical analyses

Data are presented as means ± standard error of the mean (SEM). ANOVAs, followed by Tukey *post hoc* tests, were used to analyze isolated esophageal and intestinal respirometry data, whereas repeated measures ANOVAs, followed by Tukey *post hoc* tests, were used to analyze SMR and resting metabolic rate (RMR; unfed and fully acclimated fish displaying only routine activity) data. All statistical tests were performed in R using the jamovi platform (version 0.7.5.4; jamovi project 2017).

## 3 Results

### 3.1 Whole-animal respirometry

**FIGURE 1:**
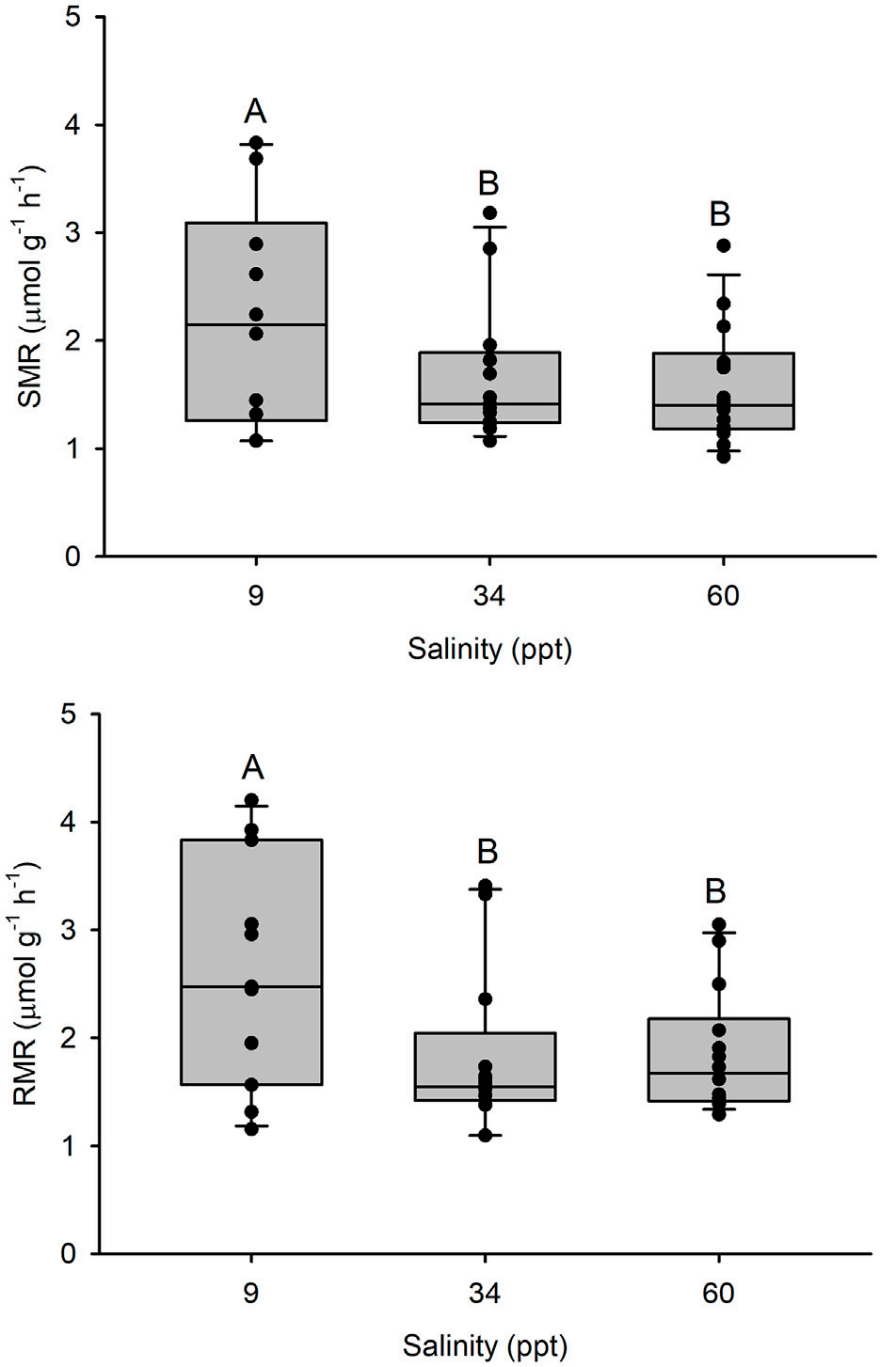
Top panel: Standard metabolic rate (SMR) and bottom panel: Resting metabolic rate (RMR) in Gulf toadfish (*Opsanus beta*) acclimated to 9, 34 or 60 ppt. Median, range, upper and lower quartile as well as individual observations (n = 10, 13, 14, respectively). Groups labelled with different letters are statistically significant different (*p* < 0.05).

SMR was 1,682 ± 180 µmol O_2_ kg^-1^ h^-1^ in 34 ppt seawater (n = 13). A higher SMR was observed in fish acclimated to 9 ppt (*p* < 0.05) while the SMR of fish acclimated to 60 ppt was not significantly different from that of 34 ppt acclimated fish (Figure 1). A similar pattern was observed for RMR with 1825 ± 208 µmol O_2_ kg^-1^ h^-1^ in 34 ppt seawater (n = 13) (*p* < 0.05).

### 3.2 Theoretical calculations for MO_2_ in esophageal and intestinal epithelia

**FIGURE 2:**
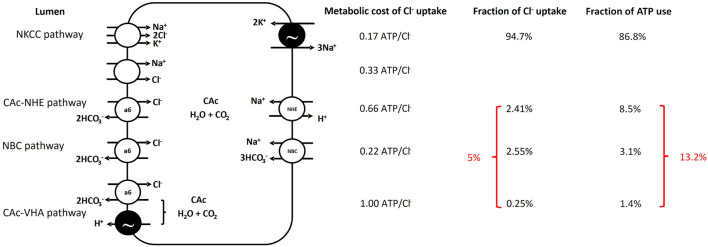
Schematic representation of Cl^−^ absorption pathways in the Gulf toadfish (and likely most marine teleosts) intestine along the theoretical ATP consumption required for Cl^−^ absorption by each pathway, the fraction of overall Cl^−^ absorption conducted by each pathway and finally the resulting fraction of overall ATP consumption related to Cl^−^ absorption by individual pathways. The NKCC2 pathway is responsible for the majority of Cl^−^ absorption but there is no evidence for involvement of the Na^+^, Cl^−^—cotransporter. The anion transport pathways are generally more metabolically costly than the NKCC2 pathway and specifically, the pathways involving hydration of endogenous CO_2_ via carbonic anhydrase are the most metabolically costly. Note that the modest contribution by anion exchange pathways to Cl^−^ absorption of 5% corresponds to 13.2% of the ATP consumption required for Cl^−^ uptake. See text and Table 1 for further information.

We estimated ATP consumption related to Cl^−^ absorption, from Cl^−^ absorption rates and various assumptions of transporter stoichiometries, to be 94.8 and 117.5 µmol ATP kg^-1^ h^-1^ for the esophagus and the intestine, respectively (Table 1). For the intestine, where at least four parallel Cl^−^ absorption pathways operate, ∼95% of Cl^−^ absorption occurs *via* the least metabolically costly NKCC2 pathway with SLC26a6 pathways accounting for the remaining ∼5% (Figure 2). The SLC26a6 mediated pathways are generally more metabolically demanding (0.22–1 mol ATP/mol Cl^−^) than the NKCC2 pathway (0.17 mol ATP/mol Cl^−^) with the CAc mediated pathways being most costly (Table 1; Figure 2). The calculated ATP consumption rates allowed us to estimate O_2_ consumption rates associated with Cl^−^ absorption to be 19 and 23.5 µmol O_2_ kg^-1^ h^-1^, for the esophagus and the intestine, respectively (Table 1; Figure 2). Whole animal SMR was 1.68 ± 180 µmol O_2_ g^-1^ h^-1^ and the esophageal and intestinal contribution to SMR is thus estimated to be 1.22% and 1.4%, respectively, for an overall contribution of osmoregulatory costs in the gastro-intestinal tract of 2.62% of SMR.

### Empirical measures of MO_2_ in isolated esophageal and intestinal tissue preparations

**FIGURE 3:**
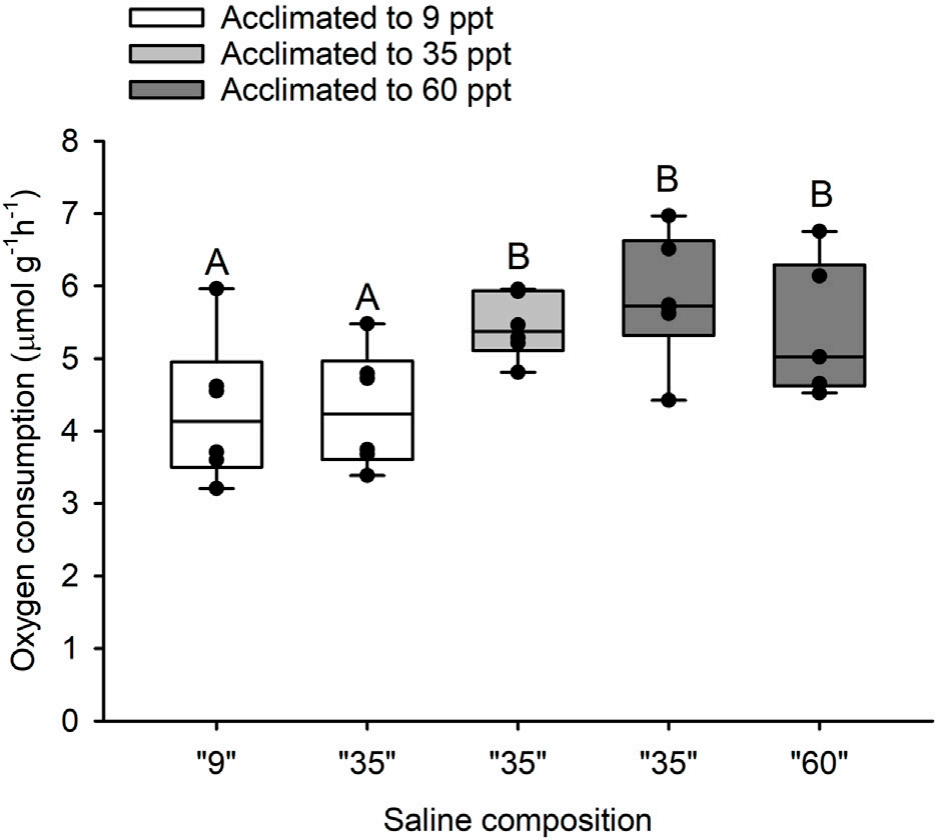
Oxygen consumption rate by isolated anterior intestinal epithelia from toadfish acclimated to 9 (open boxes), 35 (light grey) and 60 (dark grey) with different luminal salines representative of *in vivo* intestinal fluid composition at 9, 35 and 60 ppt (*X*-axis). Median, range, upper and lower quartile as well as individual observations. N = 6 for 9, 35 and 60 ppt. Groups labelled with different letters are statistically significant different (*p* < 0.05).

**FIGURE 4:**
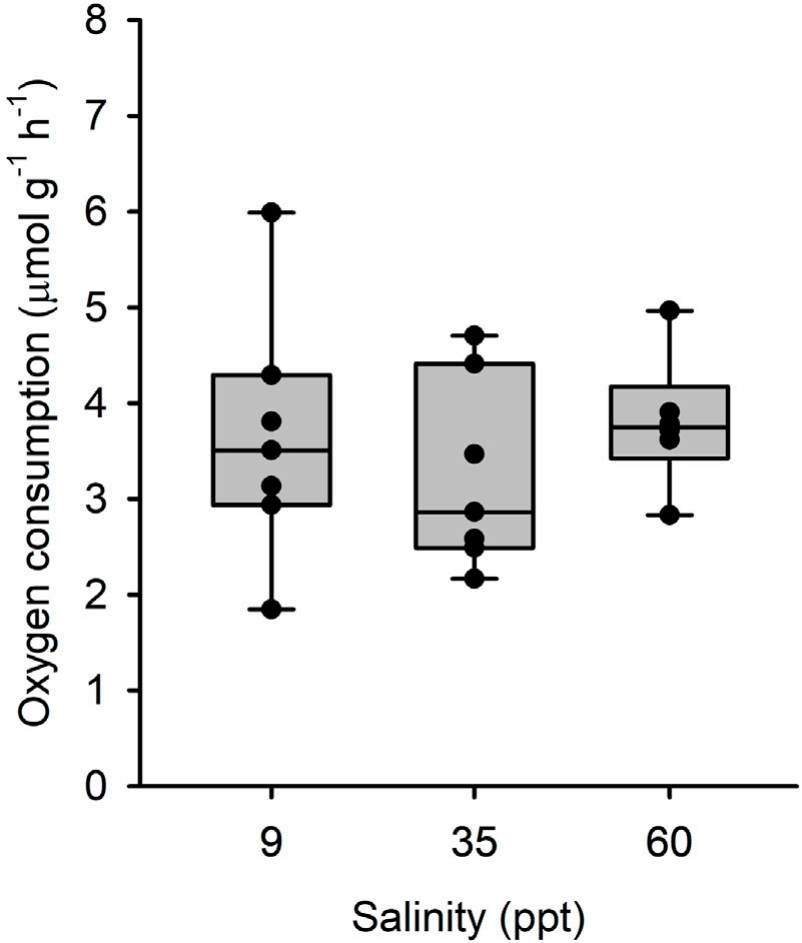
Oxygen consumption rate by isolated esophageal epithelia from toadfish acclimated to 9 (open bars), 35 (light grey) and 60 (dark grey) with different luminal salines representative of *in vivo* conditions. Median, range, upper and lower quartile as well as individual observations. N = 7, 7 and 6 for 9, 35 and 60 ppt, respectively. No statistical differences were observed.

There was a significant effect of salinity acclimation on mean intestinal MO_2_ (df = 15; F = 6.99; *p* = 0.007), where individuals acclimated to 34 ppt and 60 ppt had significantly higher mean intestinal MO_2_ (5.44 μmol g^-1^h^-1^ and 5.83 μmol g^-1^h^-1^, respectively) than those acclimated to 9 ppt (4.28 μmol g^-1^h^-1^; df = 15; t = 2.685; *p* = 0.042 and df = 15; t = −3.595; *p* = 0.007, respectively) (Figure 3). For fish acclimated to 9 ppt, luminal saline representing intestinal fluids of fish acclimated to 9 and 35 ppt caused no differences in MO_2_ of isolated intestinal tissue (Figure 3). Similarly, for fish acclimated to 60 ppt, luminal saline representing intestinal fluids of fish acclimated to 35 and 60 ppt resulted in no difference in MO_2_ of isolated intestinal tissue (Figure 3). Despite the small increase in mean MO_2_, there was no significant difference between fish acclimated to 34 ppt and 60 ppt (df = 15; t = −0.911; *p* = 0.642). There was no significant effect of salinity acclimation on mean esophageal MO_2_ between 9 ppt (4.31 μmol g^-1^h^-1^), 34 ppt (3.24 μmol g^-1^h^-1^) and 60 ppt (3.80 μmol g^-1^h^-1^; df = 17; F = 0.523; *p* = 0.602) (Figure 4).

### 3.3 Metabolic costs of osmoregulation in esophageal and intestinal tissues

The metabolic costs of osmoregulation in the intestine and esophagus were calculated from empirical data relative to mean SMR in seawater (1,682 ± 180 µmol O_2_ kg^-1^ h^-1^). For the intestine, mean intestinal MO_2_ for fish acclimated to 9 ppt was subtracted from the mean intestinal MO_2_ for fish acclimated to 34 ppt (5.44 μmol g^-1^ h^-1^—4.28 μmol g^-1^ h^-1^ **=** 1.17 μmol g^-1^ h^-1^), multiplied by mean relative (%) intestinal mass relative to whole body mass (1.17 μmol g^-1^ h^-1^ x 1.90% = 22.17 μmol kg^-1^ h^-1^), and divided by mean SMR (22.17 μmol kg^-1^ h^-1^/1,682 μmol kg^-1^ h^-1^ = 1.32% SMR). There was no difference in esophageal MO_2_ with salinity acclimation (see above). However, the primary function of the esophagus in marine teleost fish is ion transport. Thus, mean esophageal MO_2_ was multiplied by mean relative (%) esophageal mass (3.56 μmol g^-1^ h^-1^ x 0.56% = 19.89 μmol kg^-1^ h^-1^), and divided by mean SMR (19.89 μmol kg^-1^ h^-1^/1,682 μmol kg^-1^ h^-1^ = 1.18% SMR) to calculate absolute esophageal osmoregulatory costs. Thus, esophageal and intestinal osmoregulatory processes, based on isolated tissue respirometry, account for approximately 2.5% SMR.

## 4 Discussion

### 4.1 Cost of esophageal and intestinal transport

The first empirical data on metabolic cost of osmoregulation by the esophagus and intestinal epithelium of a marine teleost fish presented here suggests that 2.5% of SMR is devoted to this essential physiological function. Corresponding theoretical estimates of metabolic cost of osmoregulatory processes were possible for the gulf toadfish for which detailed information is available for the relevant ion transport pathways. These estimates predicted a cost of intestinal osmoregulatory processes of 2.6% of SMR and are thus in close agreement with our empirical data. Although isolated epithelia under Ussing chamber style conditions are generally accepted to be healthy and viable, any deviation from in vivo-like conditions would likely result in underestimates of true metabolic rates because the isolated tissue lacks primary and secondary circulation, neuroendocrine stimulation, and may not have optimal mixtures of metabolic fuel. Similarly, theoretical estimates of metabolic cost of ion transport could underestimate true metabolic cost as these calculations assume 100% efficiency of transport processes and no significant back flux of ions. However, the most parsimonious explanation for the close agreement between the two approaches seems to be that neither process is associated with significant error. Errors of the same magnitude, caused by different potential limitations, constraints on isolated tissues *versus* erroneous assumptions of transport efficiencies, on two widely distinct approaches seem less likely. We did not include the rectal epithelium in our measurements or estimates. The rectal epithelium does contribute to osmoregulation in toadfish (Ruhr et al., 2016) but represents a small fraction of length and mass compared to the intestine and its contribution to overall metabolic cost of osmoregulation is therefore modest.

The metabolic cost of intestinal osmoregulatory processes of 2.5%–2.6% of SMR is modest. However, the metabolic rate of the intestinal tissue (5.44 μmol g^-1^ h^-1^ at 22°C) is similar to previously reported values from this species (8.9 μmol g^-1^ h^-1^ at 25°C) (Taylor and Grosell, 2009) and 3.2 times higher than the corresponding mass specific whole animal metabolic rate illustrating the overall high metabolic demand of this tissue. Similar results were found for the esophageal tissue, with its mass specific metabolic rate 2.1-fold higher than the corresponding whole animal metabolic rate. Further, 21% (1.17/5.44 μmol g^-1^ h^-1^) of this high tissue specific metabolic demand is allocated to ion transport processes in the intestine, while most of the metabolic demand of the esophageal tissue appears to be associated with ion absorption. Our observations add perspective to previous work showing elevated cardiac output devoted mainly to increased gut blood flow in the euryhaline rainbow trout (*Oncorhynchus mykiss*) following acclimation to seawater (Brijs et al., 2015; Brijs et al., 2016). This increase in gut blood flow is necessary to sustain the higher metabolic demand of intestinal tissue associated with osmoregulation in seawater and allowed for normal specific dynamic action and postprandial increases in gut blood flow in seawater acclimated rainbow trout despite the increased metabolic cost associated with osmoregulation (Brijs et al., 2016).

Kirschner (Kirschner, 1993) used a similar theoretical approach to the one we employed to determine cost of osmoregulation by the intestine and the gill in seawater acclimated rainbow trout (*Oncorhyncus mykiss*) and European flounder (*Platichthys flesus*) and found that 1.4% and 3.2% of SMR could be ascribed to osmoregulatory costs by the intestine for rainbow trout and European flounder, respectively. Note that Kirschner’s original data suggests that intestinal costs are 7.4% of BMR for flounder. However, as Kirschner points out, the values for European flounder are overestimates since ion flux rates were measured at 17°C–22°C, while the BMR measurements were obtained at 10°C. Assuming a Q_10_ of 2.3 to adjust the BMR to 20°C provides an estimate of the 3.2% of BMR associated with intestinal osmoregulatory functions, as mentioned above. Kirschner assumed that all Na^+^ and Cl^−^ absorption was performed by the intestine and did not account for the component of Na^+^ and Cl^−^ absorption that occurs across the esophagus via passive transport, a factor that would tend to overestimate metabolic cost. Further, Kirschner assumed that all intestinal Na^+^ and Cl^−^ absorption occurred via NKCC. However, both European flounder (Grosell and Jensen, 1999; Grosell et al., 2005) and rainbow trout (Wilson et al., 1996; Grosell et al., 2009a) rely on anion exchange pathways in addition to the NKCC and although these pathways contribute little to overall Cl^−^ uptake, they are less energy efficient from a Cl^−^ uptake perspective. The assumption of exclusive NKCC-driven ion absorption likely represents a modest underestimation of cost of intestinal ion transport, as anion exchange pathways combined represent only ∼13% of total metabolic costs, at least in toadfish (Figure 2).

Despite these relatively minor differences between Kirschner’s original approach and ours, there is reasonable agreement among estimates of 1.4% and 3.2% of SMR for rainbow trout and European flounder, respectively (Kirschner, 1993), and our estimate of 2.6% of SMR for toadfish esophageal and intestinal osmoregulatory process. Importantly, the average of these estimates (∼2.4% of SMR) are validated by the empirical data (2.5%) in the present study.

The three intestinal ion transport pathways involving anion exchange are all energetically more costly than the NKCC pathway and although they only contribute around 5% of overall Cl^−^ uptake, they are responsible for >13% of the overall metabolic cost (Figure 2). Since the NKCC pathway is more efficient, the adaptive significance of these transport pathways does not appear to be Cl^−^ absorption. Rather, the significance of these transport pathways is likely to raise luminal HCO_3_^−^ and CO_3_^2-^ concentrations to facilitate formation of CaCO_3_ (“ichthyocarbonates”), which act to reduce luminal osmotic pressure by up to 100 mOsm (Wilson et al., 2002; Grosell et al., 2009b) thereby facilitating water absorption. Our previous work has demonstrated that intestinal water absorption, which is coupled to Na^+^ and Cl^−^ absorption, can proceed against osmotic gradients of up to 35 mOsm (Genz et al., 2011) and intestinal fluids are near isosmotic to the blood plasma. Thus, without this precipitation reaction and assuming constant plasma osmotic pressure, the osmotic pressure of the intestinal lumen would exceed that of the blood by 100 mOsm and not only prevent intestinal water absorption but also result in fluid loss into the intestine.

### 4.2 Cost of branchial and renal transport

Few studies have directly addressed the metabolic cost of osmoregulatory process in the gill despite the important role this organ plays in teleost fish osmoregulation in both freshwater and seawater. In Kirschner’s pioneering work from 1993 (Kirschner, 1993), metabolic cost of branchial ion regulatory processes was estimated using the theoretical approach to be 5.7% and 4.1% for seawater acclimated rainbow trout and European flounder, respectively, with the value for flounder derived using a temperature adjusted SMR as described in the preceding paragraph. These values are comparable but slightly higher than the only tissue specific metabolic rate measurement performed on gill tissue from seawater acclimated fish (Morgan and Iwama, 1999) indicating that 2.4% of BMR was required for branchial osmoregulatory processes but overall suggests that average branchial cost of osmoregulation in seawater teleost is, on average ∼4% of BMR.

Metabolic costs of renal osmoregulatory processes have yet to be estimated or measured as it is challenging, if not impossible, to work on the kidney in isolation. However, the agreement between empirical and theoretical estimates of osmoregulatory costs observed for the intestinal tissue in the present study is encouraging for future work aiming to determine the cost of osmoregulatory processes of the renal system. With known renal urine flow rates and composition, urinary bladder transport functions and assumptions about the secretory and absorptive processes in renal tubules, theoretical estimates of renal osmoregulatory costs should be possible although beyond the scope of this study.

### 4.3 Whole animal cost of osmoregulation

To estimate whole animal metabolic cost of osmoregulation in marine or seawater acclimated euryhaline teleost fish, it seems safe to assume that renal contributions are minor, possibly in the order of ∼1%, which when combined with an average esophageal/intestinal cost of 2.4% and an average branchial cost of 4% adds up to 7%–8% of BMR. This estimate is in close agreement with Kirschner’s cost of osmoregulation estimates, not including renal contributions, of 7.5% and 7.3% of SMR for rainbow trout and European flounder, respectively (applying the temperature correction of SMR for the European flounder as discussed above). Dozens of studies (reviewed by (Ern et al., 2014)) have attempted to determine the cost of osmoregulation by comparing metabolic rates in fish acclimated to different salinities with the expectation that the differences between metabolic rates at low or high salinities would exceed the corresponding metabolic rates at isosmotic salinities where cost of osmoregulation would approach zero and that these differences would reflect metabolic cost of osmoregulation. However, there is no consensus among the whole animal metabolic rate studies and estimates range from undetectable to >30% of BMR (Ern et al., 2014; Christensen et al., 2017; Christensen et al., 2018; Christensen et al., 2019). Similar difficulty was observed in the present study where the highest SMR and RMR was observed in fish acclimated to isosmotic 9 ppt, suggesting a negative cost of osmoregulation in seawater acclimated fish which is obviously meaningless. In our view, at least two factors contribute to difficulties in assessing metabolic costs of osmoregulation, and other vital processes, by comparing whole animal metabolic rates. First, whole animal metabolic rates vary considerably among individuals, even when care is taken to select animals of similar size and history, to standardize digestive status, and to allow for animals to settle in respirometers before measurements are initiated (Clark et al., 2013). Using the present dataset as an example, toadfish SMR in seawater of 1,682 µmol O_2_ kg^-1^ h^-1^ was associated with an SEM of ±180 (=STDEV of ± 649) µmol O_2_ kg^-1^ h^-1^, n = 13, which is equivalent to 39% variation. Assuming a whole animal metabolic cost of osmoregulation of 7.5%, *α* = 0.05, ß = 0.2 and a power of 0.8, a power analysis revealed that an n-number of 416 individuals per experimental group would be required to detect a statistically significant differences of ∼7.5% of BMR associated with osmoregulation in the present study and that of Kirschner (Kirschner, 1993). None of the studies aiming to detect cost of osmoregulation from whole animal metabolic rates, including ours, have applied replication anywhere near this requirement. Second, by nature, experiments comparing metabolic rates across salinities use euryhaline and estuarine species. For many of these species, salinity fluctuations, tidal or seasonal, may represent differences in prey abundance, predatory pressures, and reproductive status and, by association, elicit salinity induced changes in metabolic rates that are evident in estimates of SMR, but are not strictly related to the cost of osmoregulation. This second factor may explain how investigators, including us in the present study, have documented significant effects of salinity on metabolic rates even if the power of experimental design was insufficient to detect the true cost of osmoregulation.

Considering the high degree of replication required and the possible salinity-associated confounding factors that may influence whole animal metabolic rates, it seems that whole animal respirometry is impractical for studying cost of osmoregulation. Rather, isolated tissue respirometry or estimates based on ion transport rates, which were found in close agreement in the present study, are better suited to answer future questions about metabolic cost of osmoregulation. Important questions to be addressed in future studies include how osmoregulatory costs may change with climate change, size/age of the organism, and active *versus* sedentary lifestyles.

## Data Availability

The raw data supporting the conclusion of this article will be made available by the authors, without undue reservation.
